# Clinical multiplexed exome sequencing distinguishes adult oligodendroglial neoplasms from astrocytic and mixed lineage gliomas

**DOI:** 10.18632/oncotarget.2342

**Published:** 2014-08-12

**Authors:** Jane B. Cryan, Sam Haidar, Lori A. Ramkissoon, Wenya Linda Bi, David S. Knoff, Nikolaus Schultz, Malak Abedalthagafi, Loreal Brown, Patrick Y. Wen, David A. Reardon, Ian F. Dunn, Rebecca D. Folkerth, Sandro Santagata, Neal I. Lindeman, Azra H. Ligon, Rameen Beroukhim, Jason L. Hornick, Brian M. Alexander, Keith L. Ligon, Shakti H. Ramkissoon

**Affiliations:** ^1^ Department of Pathology, Brigham and Women's Hospital, Boston, MA, USA; ^2^ Department of Medical Oncology, Dana-Farber Cancer Institute, Boston, MA, USA; ^3^ Department of Pathology, Harvard Medical School, Boston, MA, USA; ^4^ Department of Neurosurgery, Brigham and Women's Hospital, Boston, MA, USA; ^5^ Department of Radiation Oncology, Dana-Farber Cancer Institute, Boston, MA, USA; ^6^ Kravis Center for Molecular Oncology & Department of Epidemiology and Biostatistics, Memorial Sloan Kettering Cancer Center, New York, New York, USA

**Keywords:** oligodendroglioma, astrocytoma, oligoastrocytoma, sequencing, IDH

## Abstract

Classifying adult gliomas remains largely a histologic diagnosis based on morphology; however astrocytic, oligodendroglial and mixed lineage tumors can display overlapping histologic features. We used multiplexed exome sequencing (OncoPanel) on 108 primary or recurrent adult gliomas, comprising 65 oligodendrogliomas, 28 astrocytomas and 15 mixed oligoastrocytomas to identify lesions that could enhance lineage classification. Mutations in *TP53* (20/28, 71%) and *ATRX* (15/28, 54%) were enriched in astrocytic tumors compared to oligodendroglial tumors of which 4/65 (6%) had mutations in *TP53* and 2/65 (3%) had *ATRX* mutations. We found that oligoastrocytomas harbored mutations in *TP53* (80%, 12/15) and *ATRX* (60%, 9/15) at frequencies similar to pure astrocytic tumors, suggesting that oligoastrocytomas and astrocytomas may represent a single genetic or biological entity. p53 protein expression correlated with mutation status and showed significant increases in astrocytomas and oligoastrocytomas compared to oligodendrogliomas, a finding that also may facilitate accurate classification. Furthermore our OncoPanel analysis revealed that 15% of *IDH1/2* mutant gliomas would not be detected by traditional IDH1 (p.R132H) antibody testing, supporting the use of genomic technologies in providing clinically relevant data. In all, our results demonstrate that multiplexed exome sequencing can support evaluation and classification of adult low-grade gliomas with a single clinical test.

## INTRODUCTION

Adult gliomas account for 20% of primary brain tumors, comprising a spectrum of tumors with varying grades (WHO Grade I-IV) and dramatic differences in patient outcomes and survival [1]. Glioblastoma (GBM, WHO Grade IV) is the most common primary malignant brain tumor of adults accounting for 54% of all gliomas with a median survival of 15 months despite surgical and chemo-radiotherapeutic intervention [2, 3]. Although large-scale systematic efforts have vastly expanded our knowledge of the underlying biology and genetics of GBM, adult lower grade gliomas (ALGGs) (WHO Grade II and III), including oligodendrogliomas, astrocytomas and mixed gliomas (oligoastrocytomas) remain less studied. Importantly several recent reports have broadened our understanding of these tumors and highlighted the utility of large scale sequencing studies in identifying clinically distinct subgroups [4, 5]. These lower grade, infiltrating gliomas represent 15% of all primary brain tumors diagnosed in adults and typically manifest in younger patients (3^rd^ and 4^th^ decades) compared to GBMs which occur later in life (5^th^-7^th^ decades) [6-9]; however ALGGs can progress to higher grade lesions with resistance to standard of care therapies including radiation and chemotherapy.

Gliomas comprise a heterogeneous group of brain tumors traditionally classified by morphologic features ascribed to normal non-neoplastic cells in the brain such as astrocytes and oligodendrocytes. The current grading criteria for gliomas rely primarily on histopathologic features. Typically WHO Grade II gliomas have moderate cellularity and nuclear atypia with low mitotic indices, whereas Grade III astrocytic tumors show increasing atypia and mitoses. Additionally anaplastic oligodendrogliomas and oligoastrocytomas can present with vascular proliferation and necrosis [10]. Distinguishing tumor lineage on histologic criteria alone is challenging as tumors frequently have overlapping morphologic features. Although immunohistochemistry is routinely used to assist in distinguishing tumor lineage, it is also not definitive or reproducible. Indeed diagnostic classification of oligoastrocytomas is associated with an inter-observer variability rate approaching 50%, demonstrating the need for objective biomarkers to aid prognostic and therapeutic decision-making [11, 12].

To support diagnosis and classification of ALGGs, significant effort has been made to identify lineage-specific molecular and genetic markers. Indeed the presence of chromosome 1p and 19q deletions (1p/19q co-deletion) determined by FISH or array comparative genomic hybridization (aCGH) is currently used to support the diagnosis of oligodendrogliomas, as this event occurs in >90% of cases [13]. Mutations in isocitrate dehydrogenase 1 and 2 genes (*IDH1/2*) have been identified in >80% of ALGGs as well as a subset of GBMs that progressed from lower grade gliomas [14, 15]. *IDH1/2* mutations are the most frequent mutations detected in lower grade gliomas and those tumors associated with *IDH1/2* mutation are reported to have better outcomes compared to wild type tumors [14]. *IDH1/2* catalyze the oxidative decarboxylation of isocitrate to produce α-ketoglutarate (α-KG). Mutant *IDH1/2* enzymes gain neomorphic functions that result in the production of the putative oncometabolite 2-Hydroxyglutarate (2HG) from α-KG; however, the precise mechanism by which *IDH1/2* mutations promote tumorigenesis remains to be elucidated including other cooperating genomic events that are required for cellular transformation [16]. The presence of *IDH1/2* mutations in both astrocytomas and oligodendrogliomas suggests that this mutation occurs early in glioma development, most likely in a stem/progenitor cell that can give rise to both cell types [14].

Distinguishing between astrocytic, oligodendroglial and mixed lineage gliomas based on morphologic and immunohistochemical features continues to be challenging. Accurate determination of lineage is essential in prognostication and treatment planning for patients. With next generation sequencing rapidly integrating into the clinical and clinical research setting, we profiled a cohort of 108 ALGGs as part of a clinical research program in a CLIA certified laboratory in order to demonstrate the utility of multiplexed exome sequencing as an adjunct to traditional methods of brain tumor classification. Furthermore we sought to determine whether targeted sequencing might reliably and simultaneously capture known mutations with prognostic significance, identify patients who may benefit from targeted therapies and help re-envision a modern classification system for ALGGs incorporating histologic and molecular data to improve inter-observer reliability for diagnosis of these challenging tumors.

## RESULTS

### *TP53* and *ATRX* mutations frequently co-occur in astrocytic and mixed lineage tumors but not in oligodendrogliomas

To map mutational signatures of ALGGs across astrocytic, mixed and oligodendroglial lineages we established a cohort of 108 tumors and performed multiplexed exome sequencing using the OncoPanel platform, which covers 275 cancer related genes. The ALGG cohort consisted of 10 diffuse astrocytomas (DA2, WHO Grade II), 18 anaplastic astrocytomas (AA3, WHO Grade III), seven oligoastrocytomas (OA2, WHO Grade II), eight anaplastic oligoastrocytomas (OA3, WHO Grade III), 44 oligodendrogliomas (O2, WHO Grade II) and 21 anaplastic oligodendrogliomas (O3, WHO Grade III).

**Figure 1:**
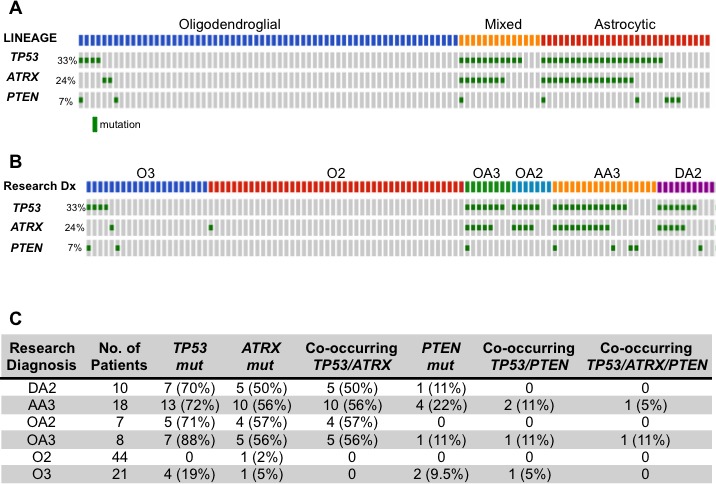
(A, B) Oncoprint diagramming *TP53*, *ATRX* and *PTEN* mutational status from 108 ALGGs categorized by lineage (A) or WHO grade (B) Gray boxes represent individual tumors and green boxes indicate presence of a mutation. (C) Table summarizing *TP53, ATRX* and *PTEN* mutations occurring singly or in combination across all tumor classes.

Tumors were classified by lineage following independent pathologist review (SHR, JBC) and then correlated with frequently mutated genes. The most common mutations across the ALGG cohort were *IDH1* and *IDH2*, which collectively occurred in 90% (97/108) of tumors suggesting that formation of these neomorphic metabolic enzymes represents an early event in gliomagenesis. Examination of lineage specific mutations showed a predominance of *TP53* mutations in astrocytic (71%, 20/28) and mixed lineage (80%, 12/15) tumors (Figure 1A, Supplemental Table 1-4). *ATRX* mutations were similarly restricted to astrocytic and mixed lineag tumors with a frequency of 54% (15/28) and 60% (9/15), respectively. Interestingly, all *ATRX* mutations co-occurred with *TP53* mutations in these tumors, suggesting that *TP53* mutations preceded *ATRX* alterations. The frequency of *TP53* and *ATRX* mutations were independent of tumor grade as both Grade II and III tumors exhibited similar mutation rates among astrocytic and mixed lineage tumors (Figure 1B, C).

In contrast *TP53* and *ATRX* mutations were rare in oligodendroglial tumors (Figure 1A, Supplemental Table 5-6). Of the 44 tumors independently assigned O2 diagnoses, only one sample contained an *ATRX* mutation, while *TP53* mutations were not detected. Comparatively, in O3 tumors we found only one sample (1/21) harbored an *ATRX* mutation while *TP53* mutations were more frequent (19%, 4/21) (Figure 1B). These findings suggest that in oligodendroglial tumors *TP53* mutations are more likely to be later events where they may function to mediate progression or resistance to therapy, while in astrocytic and mixed lineage tumors *TP53* and *ATRX* lesions are often early genetic events along with *IDH1* mutations. To further explore the implications of somatic mutations as markers of genomic progression, individual ALGG patients with multiple resections should be analyzed to compare genomic changes with histologic progression from low to high grade gliomas.

### Protein analysis of p53 and *ATRX* demonstrates differential expression across tumor lineages

**Figure 2:**
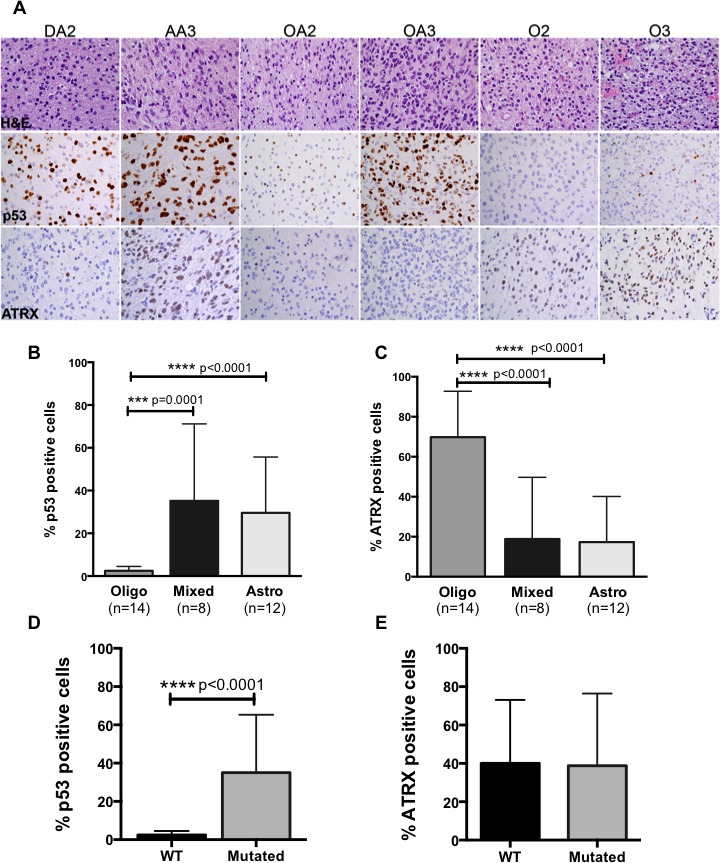
(A) Representative H&E, p53 and ATRX images from IHC analysis on tumors from each diagnostic category (B) Quantification of p53 demonstrates significant increases in nuclear positivity in astrocytic and mixed lineage tumors compared to oligodendroglial tumors, while ATRX expression is significantly increased in oligodendrogliomas (C). (D) p53 expression significantly correlated with *TP53* mutations whereas ATRX expression was independent of mutation status across ALGG cohort (E).

To determine whether p53 and ATRX protein levels correlate with mutation status or glioma lineage, we performed immunohistochemistry on a subset of patient samples. We found that p53 nuclear positivity was high in DA2, AA3 and OA3 tumors but present at low levels in oligodendroglial tumors (Figure 2A). 25% of DA2, 34% of AA3, 7% of OA2 and 63% of OA3 had high levels of p53 in the nucleus, whereas oligodendroglial tumors demonstrated significantly lower levels of nuclear p53 (2.3% in O2, 2.7% in O3) (Supplemental Figure 1A). When comparing p53 levels between grade II and III tumors within the same lineage, we found that only mixed lineage tumors showed a statistically significant (p<0.0001) increase in p53 levels in higher-grade tumors.

Consistent with high frequencies of wildtype *ATRX* in oligodendroglial tumors, ATRX protein expression was high in these tumors while astrocytic and mixed lineage tumors in which *ATRX* is frequently mutated (loss of function events) exhibited low protein levels. ATRX was present in the nucleus of 61% and 79% of O2 and O3, respectively, but in the nucleus of only 3%, 31%, 1% and 36% of DA2, AA3, OA2 and OA3, respectively (Supplemental Figure 1B). Our findings also showed ATRX protein levels were significantly increased in grade III tumors compared to grade II across all lineages.

Correlative analysis between glioma lineage and protein levels showed p53 levels were significantly higher in astrocytic and mixed lineage tumors compared to oligodendroglial tumors while the opposite was true for ATRX; oligodendroglial tumors expressed higher ATRX levels compared to astrocytic and mixed lineage tumors (Figure 2B, C). Furthermore when we compared p53 levels to *TP53* mutational status we found that tumors with *TP53* mutations were significantly more likely to express p53 protein (Figure 2D). In contrast, ATRX protein expression was independent of *ATRX* mutation status (Figure 2E).

Taken together these findings suggest that protein analysis of p53 and ATRX in ALGGs support our hypothesis that astrocytic and mixed lineage tumors depend on loss of p53 and ATRX functions while oligodendroglial tumors are driven by alternate mechanism(s).

### Spectrum of *IDH* mutations in adult lower grade gliomas reveals the utility of exome sequencing

**Figure 3:**
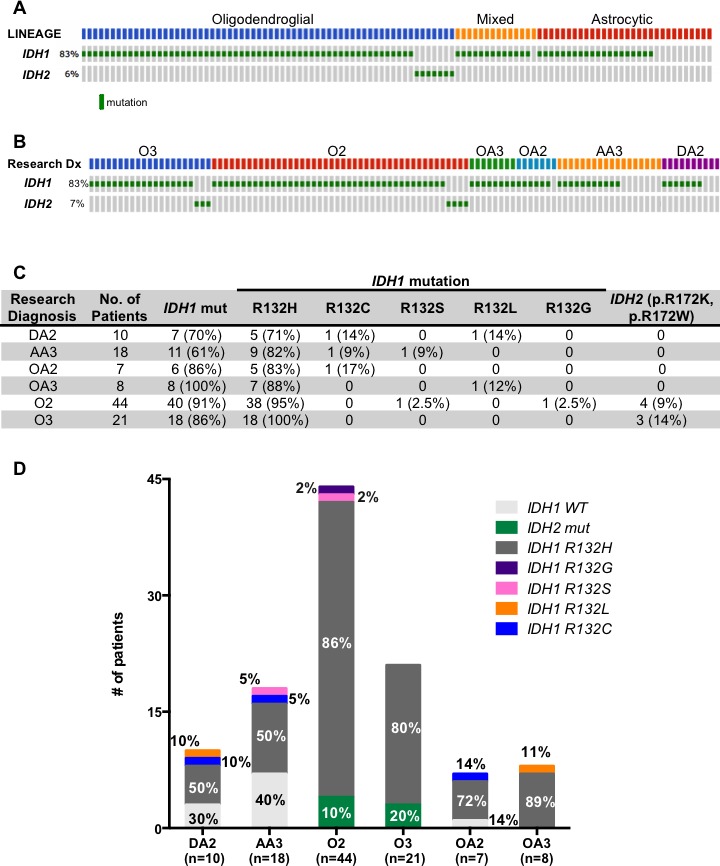
(A, B) Oncoprint diagramming *IDH1* and *IDH2* mutational status from 108 ALGGs categorized by lineage (A) or WHO grade (B) (C) Table summarizing *IDH1* and *IDH2* mutations across all tumor classes. (D) Graph representing frequency of *IDH1* and *IDH2* variants in each diagnostic category.

As previously described our cohort of ALGGs demonstrated a high frequency of *IDH1/2* mutations; however the spectrum of mutations was diverse and highlighted the need for integration of sequencing based assays into routine diagnostics. At present, the standard method for detecting *IDH1* mutations is by immunohistochemistry (IHC) using an antibody specific for the *IDH1* p.R132H variant. Since the OncoPanel sequencing platform includes *IDH1* and *IDH2*, we sought to characterize the spectrum of *IDH1/2* mutations given the important clinical and prognostic implications of *IDH1/2* mutations in gliomas. As demonstrated in Figure 3A, 90% (97/108) of tumors in our cohort harbored either an *IDH1* or *IDH2* mutation. When analyzed by lineage, 100% (65/65) of pure oligodendroglial tumors contained *IDH1/2* mutations while 64.3% and 93.3% of astrocytic and mixed lineage tumors were positive for *IDH1/2* mutations, respectively (Figure3C). Interestingly *IDH2* mutations were restricted to oligodendroglial tumors with a similar distribution between O2 and O3. The most common mutation identified was *IDH1* p.R132H, which occurred in the majority of tumors independent of lineage or grade (Figure 3C, D); however we found that over 13.9% (15/108) of tumors harbored non-*IDH1* p.R132H variants or *IDH2* mutations, which would not be detected by traditional IHC analysis. Among DA2s *IDH1* p.R132L or p.R132C accounted for 20% of *IDH1* mutations (n=1, 1 respectively) while 11% of AA3s harbored *IDH1* p.R132C or p.R132S mutations. This was similar for mixed lineage tumors that presented with 13.3% (2/15) *IDH1* p.R132L and p.R132C mutations. Furthermore despite the high frequency of *IDH1* p.R132H mutations detected in oligodendroglial tumors, we found that 13.6% (6/44) of O2s had *IDH2*, *IDH1* p.R132G or *IDH1* p.R132S mutations. Similarly 14.3% (3/21) of O3 tumors in our cohort harbored *IDH2* p.R172K or p.R172W mutations. In sum, a total of 13.9% (15/108) *IDH1/2* mutant tumors in our ALGG cohort would have eluded detection if relying solely on traditional *IDH1* p.R132H immunohistochemistry.

### *IDH1/2* negative adult low-grade gliomas enrich for EGFR mutations and amplifications

**Figure 4:**
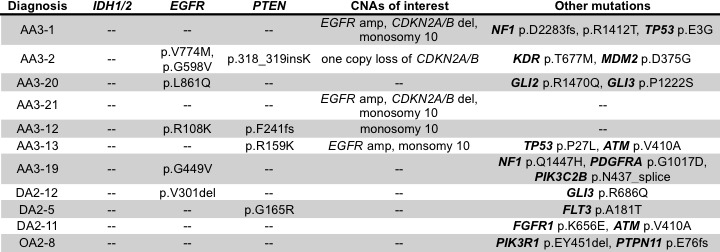
Table summarizing copy number alterations and mutations in *IDH1/2* wildtype ALGGs

As expected *IDH* mutations were the most prevalent lesion in our ALGG cohort; however, 10% of tumors (11/108) were negative for *IDH1/2* mutations, including three DA2s, one OA2 and seven AA3s. To determine whether these tumors harbored unique or unifying lesions, we expanded our analysis to assess copy number alterations as well as somatic mutations. Of the seven AA3 tumors, three contained *EGFR* amplification with monosomy 10 or *CDKN2A/B* deletion, a pattern more typical of GBM than ALGG suggesting that these tumors may in fact be more clinically aggressive than typical AA3s or under-sampled with respect to the overall features of the tumor (Figure 4). The remaining four AA3s lacked *EGFR* amplification but were positive for *EGFR* mutations (p.V774M, p.G598V, p.L861Q, p.R108K, p.G449V). Similarly two DA2s harbored *EGFR* (p.V301del) and *PTEN* (p.G165R) mutations respectively, whereas the third DA2 harbored the oncogenic *FGFR1* p.K656E mutation, which has recently been implicated in pediatric GBMs and pontine gliomas [17, 18]. The single *IDH1/2* wildtype OA2 showed *PIK3R1* p.EY451del, and a frameshift mutation (p.E76fs) in the cancer associated phosphatase *PTPN11* gene. These analyses highlight the utility of multiplexed exome sequencing in detecting tumors most likely to follow a more aggressive clinical path as well as potential therapeutic targets.

## DISCUSSION

**Figure 5:**
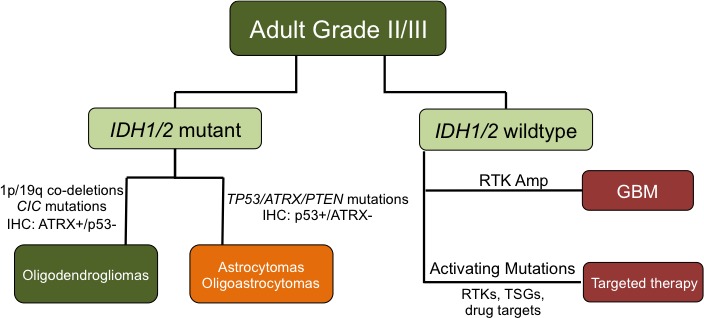
Schematic proposing an integrated classification for distinguishing ALGGs utilizing *IDH1/2*, *TP53*, *ATRX* and *EGFR* mutation status

Clinical use of next-generation sequencing technologies represents a complementary data stream that can serve as an aid to improve the reproducibility of diagnosis and classification of primary brain tumors. Here we report our findings from analyzing multiplexed exome sequencing data generated in a CLIA-certified clinical laboratory for integration into diagnostic pathology. Traditionally, the diagnosis of ALGGs has relied solely on W.H.O. criteria for morphologic features identified by light microscopy. While advances in immunohistochemical and molecular assays have supplemented this gold standard approach, we demonstrate how incorporation of sequencing data could readily improve tumor diagnosis and classification, thereby making it the next step in the evolution of pathological classification of brain tumors (Figure 5).

Our analysis of 108 ALGGs encompassing astrocytic, mixed and oligodendroglial lineage tumors revealed that *TP53* mutations were most frequent in astrocytic and mixed lineage tumors but were rarely present in oligodendroglial tumors and when present were only seen in anaplastic oligodendrogliomas, consistent with previous studies [19]. These findings suggest that early events in the formation of oligodendroglial tumors include *IDH1/2* mutations in association with 1p/19q co-deletion, *FUBP1* and *CIC* mutations but not *TP53* mutations. When present in anaplastic oligodendrogliomas, *TP53* mutations may represent a marker of progression and/or resistance to therapies, which is supported by previous studies showing that *TP53* mutations in malignant gliomas are a primary mechanism leading to radio-resistance [20]. Similarly, *ATRX* mutations were also enriched in astrocytic and mixed lineage tumors but only 2/65 oligodendroglial tumors contained *ATRX* mutations. When *ATRX* mutations were present in astrocytic and mixed lineage gliomas, they always (100%) co-occurred with *TP53* mutations. These findings suggest that (1) *TP53* mutations occur prior to developing *ATRX* mutations in astrocytic and mixed lineage gliomas and, (2) *TP53* mutations predispose a tumor to developing *ATRX* mutations, which may further accelerate tumorigenesis.

To further assess the relevance of p53 and ATRX as distinguishing biomarkers among ALGGs we performed immunohistochemistry on a subset of tumors and showed that p53 is expressed at significantly lower levels in oligodendroglial tumors compared to astrocytic and mixed gliomas. In fact, *TP53* mutated tumors significantly correlated with increased protein expression, which is consistent with high protein expression in astrocytic and mixed lineage tumors. Conversely oligodendroglial tumors demonstrated significantly increased ATRX protein expression compared to astrocytic and mixed lineage tumors, however *ATRX* mutations were not correlated with ATRX expression, consistent with previously published studies [21, 22]. Together these findings suggest that IHC analysis will provide valuable, real time adjuncts to pathologic classification of ALGGs wherein oligodendrogliomas are more likely to have high ATRX protein levels in conjunction with low p53 expression and positive IDH1 p.R132H staining.

We investigated the spectrum of *IDH1/2* mutations in ALGGs given their critical role as prognostic indicators for a more favorable clinical course compared to *IDH* wildtype gliomas. The current standard-of-care assay in clinical labs is to perform IHC for IDH1 p.R132H, the most common variant among *IDH1* mutations. In our study we demonstrated that among the 97 *IDH1/2* mutated ALGGs in our cohort, 17% (15/97) would not have been detected by IHC against IDH1 p.R132H. In fact, 10% of O2s and 20% of O3s were positive for *IDH2* mutations and negative for *IDH1* mutations. Given the clinical value of identifying *IDH1/2* mutated gliomas, our findings provide a compelling reason to advance IDH1 p.R132H protein negative ALGGs for exome or targeted sequencing of *IDH1/2*.

Although the prevalence of *IDH1/2* mutations among ALGGs was high (90%), it suggests that for the remaining 10% of *IDH1/2* wildtype tumors an alternate mechanism might be attributed to gliomagenesis. In fact, among the seven *IDH1/2* wildtype AA3s, 3 harbored polysomy 7 with *EGFR* amplification and monosomy 10, a pattern of genomic aberrations more consistent with GBM rather than AA3. These findings suggest that a subset of *IDH1/2* wildtype AA3 tumors may represent (1) under sampled GBMs or (2) incipient GBMs that have not yet developed the morphologic criteria (microvascular proliferation and/or necrosis) sufficient to be designated as WHO Grade IV. The four other *IDH1/2* wildtype AA3s each harbored varying *EGFR* mutations in combination with other gene variants (e.g. *PTEN*, *TP53*, *PDGFRA*) suggesting an alternate mechanism to gliomagenesis.

The judicious integration of next-generation sequencing analysis into the classification of brain tumors based on our studies represents an opportunity to improve diagnosis and reproducibility of glioma lineage classification across institutions. In fact our study suggests tighter correlations with histology are possible than in prior studies where histologic assignments may have been less strict or may not have involved re-review of diagnoses included in the genomic analysis. The findings highlighted in this study provide insights into how this new integrated schema would work for ALGGs by combining traditional histopathology, molecular and sequencing data to classify ALGGs (Figure 5). Based on our data, following histopathology review, tumors classified along the ALGG spectrum including WHO Grades II and III astrocytomas, oligodendrogliomas and mixed tumors could be initially divided based on *IDH1/2* mutations into mutant and wildtype groups. For institutions without the ability to perform exome sequencing, there still remains a critical need to determine *IDH1/2* mutational status among ALGG patients; therefore rapid PCR assays, targeting *IDH1* codon 132 and *IDH2* codon 172, could serve as a feasible alternative.

As the majority of ALGGs are likely to be *IDH1/2* mutated, oligodendroglial tumors can be readily identified by the presence of 1p/19q co-deletion, mutations in *CIC* and *FUBP1*, and high ATRX with low p53 protein expression. Astrocytomas and oligoastrocytomas would most often harbor mutations in *TP53*, *ATRX* and *PTEN* and show high p53 with low ATRX protein expression. In fact, based on the diagnostic challenges distinguishing astrocytomas from oligoastrocytomas, our findings suggested that these tumors are likely morphologic variants belonging in a single category. This hypothesis is supported by the nearly identical frequencies and patterns of *TP53, ATRX* and *PTEN* mutations; however a greater number of tumors, especially oligoastrocytomas, will need to be analyzed in order to fully define the relationship between astrocytomas and mixed gliomas. Based on our findings astrocytomas and mixed tumors could be classified simply as Diffuse Glioma (*IDH1/2* mutant) WHO II or Anaplastic Diffuse Glioma (*IDH1/2* mutant) WHO Grade III based on traditional glioma grading criteria.

ALGGs found to lack *IDH1/2* mutations need to be thoroughly re-investigated with integration of clinical, surgical and neuroimaging data to ensure adequate sampling of the patients’ tumors. Three of 7 AA3s in our cohort showed evidence of *EGFR* amplification, monosomy 10 and *CDKN2A/B* deletion a pattern more typical of GBM rather than ALGG. Therefore, as sequencing and genomic data become integrated into routine pathology diagnostics, such tumors may need to be evaluated as to whether molecular upgrading to GBM is warranted even when overt necrosis or microvascular proliferation are not present on light microscopy. Alternatively, *IDH1/2* wildtype ALGGs that lack a genomic GBM signature should be evaluated for other potential oncogenic drivers, including mutations such as *FGFR1* p.K656E, which has been identified in pediatric gliomas. As such, these patients may benefit from early intervention with targeted inhibitors in combination with traditional therapies such as temozolomide.

In addition to providing complimentary data for ALGG classification, targeted exome sequencing may also prove valuable in guiding therapeutic decision making. With open clinical trials for *IDH1* mutant glioma patients, determining *IDH1* mutational status for ALGG patients is critical for satisfying trial entry criteria. Similarly, as *IDH2* inhibitors currently in trial for hematologic malignancies open for glioma patients, sequencing remains the only method of identifying eligible patients. Furthermore as studies have shown that *IDH1/2* mutant gliomas have a favorable prognosis compared to their wildtype counterparts, excluding or controlling for these patients in GBM clinical trials will be critical when analyzing results to ensure that *IDH1/2* mutant GBM patients do not confound interpretation of drug effectiveness on patient survival.

Our study highlights that targeted exome sequencing of ALGGs performed in the clinical setting and CLIA-certified environment provides valuable data, which can be used in lineage classification, to refine diagnoses made by light microscopy and provide mutational data, which may be valuable in selecting appropriate targeted therapies.

## METHODS

### Patient Selection

Analysis of data generated from tumor specimens and clinical variables was conducted following approval from the Dana-Farber/Brigham and Women's Cancer Center (DF/BWCC) Institutional Review Board (IRB). Genotyping data from clinical OncoPanel testing reports was obtained from the medical record under a consented research protocol approved by the DF/BWCC IRB (11-104). All sequencing assays were performed within the Molecular Diagnostics Division of the Brigham and Women's Hospital Center for Advanced Molecular Diagnostics, a CLIA-certified laboratory environment.

All tumors underwent central histopathologic re-review using World Health Organization (WHO) criteria by two neuropathologists (J.B.C. and S.H.R.). Diagnosis of oligoastrocytomas required histologic evidence of astrocytic and oligodendroglial components, with the minor population representing >30% of tumor cells. In total, 108 brain tumors including 10 diffuse astrocytomas, 18 anaplastic astrocytomas, 44 oligodendrogliomas grade II, 21 oligodendrogliomas grade III, 7 oligoastrocytomas grade II, and 8 oligoastrocytomas grade III were analyzed and included for this study. All tumors were analyzed for the presence of 1p/19q co-deletion detected by FISH, aCGH or copy number analysis from exome data. aCGH and FISH analyses were performed in the Cytogenetics Division of the Brigham and Women's Hospital Center for Advanced Molecular Diagnostics, a CLIA-certified laboratory environment

### OncoPanel

DNA was isolated from 5-10 5μm FFPE slides containing at least 50% tumor nuclei using routine extraction methods previously described [23]. Somatic mutations in tumor DNA were detected using the exome-sequencing platform OncoPanel (Illumina HiSeq) in the DF/BWCC CLIA-certified laboratory [24]. The OncoPanel assay detects mutations in 275 different cancer genes. The average time from tissue submission to report of data was six weeks. Figures 1, 3 and Supplemental Figure 1 were developed using a local instance of the MSKCC cBioPortal for Cancer Genomics software [25]. Oncopanel mutation datasets were reformatted and imported into a mysql database using cBio's importer tool and then visualized using cBio's OncoPrint module [25].

### Immunohistochemistry

Diaminobenzidine (DAB), brightfield staining was performed according to standard protocols on five-micron thick paraffin sections [26]. Antigens were retrieved using heat and 10 mM sodium citrate buffer (pH 6.0). The following primary antibodies were utilized: p53 (ImmunoTech #1767), ATRX (Sigma, HPA001906), IDH1(R132H) (Dianova, DIA-H05). Counterstaining for nuclei was performed using Mayer's hematoxylin stain and coverslips were mounted with Permount (Fisher Scientific).

## SUPPLEMENTARY MATERIAL, FIGURE AND TABLES



## References

[1] Ostrom QT, Gittleman H, Farah P, Ondracek A, Chen Y, Wolinsky Y, Stroup NE, Kruchko C, Barnholtz-Sloan JS (2013). CBTRUS statistical report: Primary brain and central nervous system tumors diagnosed in the United States in 2006-2010. Neuro Oncol.

[2] Stupp R, Mason WP, van den Bent MJ, Weller M, Fisher B, Taphoorn MJ, Belanger K, Brandes AA, Marosi C, Bogdahn U, Curschmann J, Janzer RC, Ludwin SK, Gorlia T, Allgeier A, Lacombe D (2005). Radiotherapy plus concomitant and adjuvant temozolomide for glioblastoma. The New England journal of medicine.

[3] Stupp R, Hegi ME, Mason WP, van den Bent MJ, Taphoorn MJ, Janzer RC, Ludwin SK, Allgeier A, Fisher B, Belanger K, Hau P, Brandes AA, Gijtenbeek J, Marosi C, Vecht CJ, Mokhtari K (2009). Effects of radiotherapy with concomitant and adjuvant temozolomide versus radiotherapy alone on survival in glioblastoma in a randomised phase III study: 5-year analysis of the EORTC-NCIC trial. Lancet Oncol.

[4] Kannan K, Inagaki A, Silber J, Gorovets D, Zhang J, Kastenhuber ER, Heguy A, Petrini JH, Chan TA, Huse JT (2012). Whole-exome sequencing identifies ATRX mutation as a key molecular determinant in lower-grade glioma. Oncotarget.

[5] Liu XY, Gerges N, Korshunov A, Sabha N, Khuong-Quang DA, Fontebasso AM, Fleming A, Hadjadj D, Schwartzentruber J, Majewski J, Dong Z, Siegel P, Albrecht S, Croul S, Jones DT, Kool M (2012). Frequent ATRX mutations and loss of expression in adult diffuse astrocytic tumors carrying IDH1/IDH2 and TP53 mutations. Acta neuropathologica.

[6] Sanai N, Chang S, Berger MS (2011). Low-grade gliomas in adults. Journal of neurosurgery.

[7] Grier JT, Batchelor T (2006). Low-grade gliomas in adults. Oncologist.

[8] Olson JD, Riedel E, DeAngelis LM (2000). Long-term outcome of low-grade oligodendroglioma and mixed glioma. Neurology.

[9] Shafqat S, Hedley-Whyte ET, Henson JW (1999). Age-dependent rate of anaplastic transformation in low-grade astrocytoma. Neurology.

[10] Louis DN, Ohgaki H, Wiestler OD, Cavenee WK, Burger PC, Jouvet A, Scheithauer BW, Kleihues P (2007). The 2007 WHO classification of tumours of the central nervous system. Acta Neuropathol.

[11] Castillo MS, Davis FG, Surawicz T, Bruner JM, Bigner S, Coons S, Bigner DD (2004). Consistency of primary brain tumor diagnoses and codes in cancer surveillance systems. Neuroepidemiology.

[12] Aldape K, Simmons ML, Davis RL, Miike R, Wiencke J, Barger G, Lee M, Chen P, Wrensch M (2000). Discrepancies in diagnoses of neuroepithelial neoplasms: the San Francisco Bay Area Adult Glioma Study. Cancer.

[13] Reifenberger G, Louis DN (2003). Oligodendroglioma: toward molecular definitions in diagnostic neuro-oncology. Journal of neuropathology and experimental neurology.

[14] Yan H, Parsons DW, Jin G, McLendon R, Rasheed BA, Yuan W, Kos I, Batinic-Haberle I, Jones S, Riggins GJ, Friedman H, Friedman A, Reardon D, Herndon J, Kinzler KW, Velculescu VE (2009). IDH1 and IDH2 mutations in gliomas. N Engl J Med.

[15] Hartmann C, Meyer J, Balss J, Capper D, Mueller W, Christians A, Felsberg J, Wolter M, Mawrin C, Wick W, Weller M, Herold-Mende C, Unterberg A, Jeuken JW, Wesseling P, Reifenberger G (2009). Type and frequency of IDH1 and IDH2 mutations are related to astrocytic and oligodendroglial differentiation and age: a study of 1,010 diffuse gliomas. Acta neuropathologica.

[16] Zhao S, Lin Y, Xu W, Jiang W, Zha Z, Wang P, Yu W, Li Z, Gong L, Peng Y, Ding J, Lei Q, Guan KL, Xiong Y (2009). Glioma-derived mutations in IDH1 dominantly inhibit IDH1 catalytic activity and induce HIF-1alpha. Science.

[17] Jones DT, Hutter B, Jager N, Korshunov A, Kool M, Warnatz HJ, Zichner T, Lambert SR, Ryzhova M, Quang DA, Fontebasso AM, Stutz AM, Hutter S, Zuckermann M, Sturm D, Gronych J (2013). Recurrent somatic alterations of FGFR1 and NTRK2 in pilocytic astrocytoma. Nat Genet.

[18] Fontebasso AM, Papillon-Cavanagh S, Schwartzentruber J, Nikbakht H, Gerges N, Fiset PO, Bechet D, Faury D, De Jay N, Ramkissoon LA, Corcoran A, Jones DT, Sturm D, Johann P, Tomita T, Goldman S (2014). Recurrent somatic mutations in ACVR1 in pediatric midline high-grade astrocytoma. Nat Genet.

[19] Okamoto Y, Di Patre PL, Burkhard C, Horstmann S, Jourde B, Fahey M, Schuler D, Probst-Hensch NM, Yasargil MG, Yonekawa Y, Lutolf UM, Kleihues P, Ohgaki H (2004). Population-based study on incidence, survival rates, and genetic alterations of low-grade diffuse astrocytomas and oligodendrogliomas. Acta neuropathologica.

[20] Mehta S, Huillard E, Kesari S, Maire CL, Golebiowski D, Harrington EP, Alberta JA, Kane MF, Theisen M, Ligon KL, Rowitch DH, Stiles CD (2011). The central nervous system-restricted transcription factor Olig2 opposes p53 responses to genotoxic damage in neural progenitors and malignant glioma. Cancer Cell.

[21] Wiestler B, Capper D, Holland-Letz T, Korshunov A, von Deimling A, Pfister SM, Platten M, Weller M, Wick W (2013). ATRX loss refines the classification of anaplastic gliomas and identifies a subgroup of IDH mutant astrocytic tumors with better prognosis. Acta neuropathologica.

[22] Abedalthagafi M, Phillips JJ, Kim GE, Mueller S, Haas-Kogen DA, Marshall RE, Croul SE, Santi MR, Cheng J, Zhou S, Sullivan LM, Martinez-Lage M, Judkins AR, Perry A (2013). The alternative lengthening of telomere phenotype is significantly associated with loss of ATRX expression in high-grade pediatric and adult astrocytomas: a multi-institutional study of 214 astrocytomas. Modern pathology : an official journal of the United States and Canadian Academy of Pathology, Inc.

[23] MacConaill LE, Campbell CD, Kehoe SM, Bass AJ, Hatton C, Niu L, Davis M, Yao K, Hanna M, Mondal C, Luongo L, Emery CM, Baker AC, Philips J, Goff DJ, Fiorentino M (2009). Profiling critical cancer gene mutations in clinical tumor samples. PloS one.

[24] Wagle N, Berger MF, Davis MJ, Blumenstiel B, Defelice M, Pochanard P, Ducar M, Van Hummelen P, Macconaill LE, Hahn WC, Meyerson M, Gabriel SB, Garraway LA (2012). High-throughput detection of actionable genomic alterations in clinical tumor samples by targeted, massively parallel sequencing. Cancer discovery.

[25] Cerami E, Gao J, Dogrusoz U, Gross BE, Sumer SO, Aksoy BA, Jacobsen A, Byrne CJ, Heuer ML, Larsson E, Antipin Y, Reva B, Goldberg AP, Sander C, Schultz N (2012). The cBio cancer genomics portal: an open platform for exploring multidimensional cancer genomics data. Cancer discovery.

[26] Ligon KL, Alberta JA, Kho AT, Weiss J, Kwaan MR, Nutt CL, Louis DN, Stiles CD, Rowitch DH (2004). The oligodendroglial lineage marker OLIG2 is universally expressed in diffuse gliomas. Journal of neuropathology and experimental neurology.

