# Supplementary material for: Clinical multiplexed exome sequencing distinguishes adult oligodendroglial neoplasms from astrocytic and mixed lineage gliomas
Source: Oncotarget. 2014 Aug 12;5(18):8083–92. doi: 10.18632/oncotarget.2342 (PMC4226668; doi:10.18632/oncotarget.2342)
Supplement: Supplementary file 1 [file oncotarget-05-8083-s001.pdf]

Clinical multiplexed exome sequencing distinguishes adult oligodendroglial neoplasms from astrocytic and mixed lineage gliomas

Supplementary Material

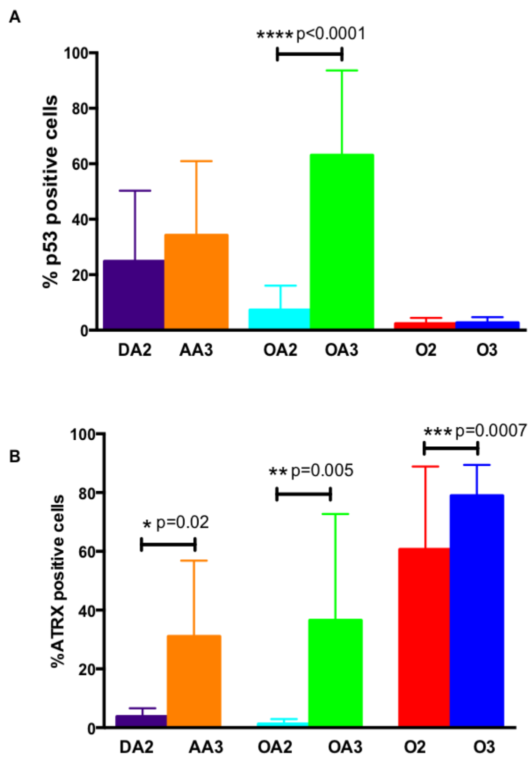

Supplemental Figure 1: (**A**) Quantification of p53 expression by tumor lineage and grade. Only oligoastrocytomas showed a significant increase in p53 nuclear positivity with increasing grade. (**B**) Quantification of ATRX expression by tumor lineage and grade revealed a significant increase in nuclear positivity with increasing grade across all lineages.

Supplemental Table 1: 1p/19q status, *IDH1*, *IDH2*, *TP53*, *ATRX* and *PTEN* mutations in individual DA2 samples.

Sample ID	1p/19q status	*IDH1*	*IDH2*	*TP53*	*ATRX*	*PTEN*
DA2-1	intact	p.R132H	--	p.R248Q	p.E1702*	--
DA2-2	intact	p.R132H	--	p.Y163H	--	--
DA2-3	intact	p.R132C	--	p.I195F	p.K971fs	--
DA2-4	intact	--	--	--	--	--
DA2-5	intact	--	--	--	--	p.G165R
DA2-6	intact	p.R132H	--	p.R209fs*6	p.R808*	--
DA2-7	intact	p.R132H	--	p.R213*	--	--
DA2-8	intact	p.R132L	--	p.R248W	p.K971fs	--
DA2-9	intact	p.R132H	--	p.R273C	p.F2113fs	--
DA2-10	intact	--	--	--	--	--

Supplemental Table 2: 1p/19q status, *IDH1*, *IDH2*, *TP53*, *ATRX* and *PTEN* mutations in individual AA3 samples.

Sample ID	1p/19q status	*IDH1*	*IDH2*	*TP53*	*ATRX*	*PTEN*
AA3-1	intact	--	--	p.E3G	--	--
AA3-2	intact	--	--	--	--	p.318_319insK
AA3-3	intact	p.R132H	--	p.R273C	p.R781*	--
AA3-4	intact	--	--	--	--	--
AA3-5	intact	p.R132H	--	p.A138V<br>p.P152fs*18	p.R2197C	--
AA3-6	intact	p.R132H	--	p.R273C	--	p.K425fs
AA3-7	intact	p.R132H	--	p.R273C	p.E2265A<br>p.E2277A	--
AA3-8	intact	p.R132H	--	p.C242Y	p.L1602*	--
AA3-9	intact	--	--	--	--	--
AA3-10	intact	p.R132H	--	p.S241F	p.D789V	--
AA3-11	intact	p.R132C	--	p.R273C	p.S1418fs	--
AA3-12	intact	--	--	--	--	p.F241fs
AA3-13	intact	--	--	p.P27L	--	p.R159K
AA3-14	intact	--	--	--	--	--
AA3-15	intact	p.R132H	--	p.R273C	--	--
AA3-16	intact	p.R132S	--	p.R273C	p.R418*	p.I135fs
AA3-17	intact	p.R132H	--	p.K132R	p.S1387*	--
AA3-18	intact	p.R132H	--	p.R175H<br>p.R213Q	p.R808*<br>p.K1332fs	--

Supplemental Table 3: 1p/19q status, *IDH1*, *IDH2*, *TP53*, *ATRX* and *PTEN* mutations in individual OA2 samples.

Sample ID	1p/19q status	*IDH1*	*IDH2*	*TP53*	*ATRX*	*PTEN*
OA2-1	intact	--	--	--	--	--
OA2-2	intact	p.R132H	--	p.G245S	p.R1426*	--
OA2-3	intact	p.R132H	--	p.R273C	--	--
OA2-4	intact	p.R132C	--	p.R306*	p.K1332fs	--
OA2-5	intact	p.R132H	--	p.R175H<br>p.C242S	p.R418*	--
OA2-6	intact	p.R132H	--	p.P152R<br>p.M246L	p.K2283splice	--
OA2-7	intact	p.R132H	--	--	--	--

Supplemental Table 4: 1p/19q status, *IDH1*, *IDH2*, *TP53*, *ATRX* and *PTEN* mutations in individual OA3 samples.

Sample ID	1p/19q status	*IDH1*	*IDH2*	*TP53*	*ATRX*	*PTEN*
OA3-1	intact	p.R132L	--	p.R273C	p.R781*	--
OA3-2	intact	p.R132H	--	p.Y205C<br>p.M237I	--	--
OA3-3	co-del	p.R132H	--	p.P190L	p.G1937E	p.P231S
OA3-4	co-del	p.R132H	--	--	--	--
OA3-5	intact	p.R132H	--	p.R213Q	p.Y2083C	--
OA3-6	intact	p.R132H	--	p.E258Q	--	--
OA3-7	intact	p.R132H	--	p.R273C	p.R1426*	--
OA3-8	intact	p.R132H	--	p.W53*<br>p.L194R	p.E935*<br>p.E884D	--

Supplemental Table 5: 1p/19q status, *IDH1, IDH2, TP53, ATRX* and *PTEN* mutations in individual O2 samples.

Sample ID	1p/19q status	*IDH1*	*IDH2*	*TP53*	*ATRX*	*PTEN*
O2-1	co-del	p.R132H	--	--	--	--
O2-2	co-del	p.R132H	--	--	--	--
O2-3	co-del	p.R132H	--	--	--	--
O2-4	co-del	--	p.R172K	--	--	--
O2-5	co-del	p.R132H	--	--	--	--
O2-6	co-del	p.R132H	--	--	--	--
O2-7	co-del	p.R132S	--	--	p.D1051E	
O2-8	co-del	p.R132H	--	--	--	--
O2-9	co-del	p.R132H	--	--	--	--
O2-10	co-del	p.R132H	--	--	--	--
O2-11	co-del	p.R132H	--	--	--	--
O2-12	co-del	p.R132H	--	--	--	--
O2-13	co-del	p.R132H	--	--	--	--
O2-14	co-del	p.R132H	--	--	--	--
O2-15	co-del	p.R132H	--	--	--	--
O2-16	co-del	p.R132H	--	--	--	--
O2-17	co-del	p.R132H	--	--	--	--
O2-18	co-del	p.R132H	--	--	--	--
O2-19	co-del	p.R132H	--	--	--	--
O2-20	co-del	p.R132H	--	--	--	--
O2-21	co-del	p.R132H	--	--	--	--
O2-22	co-del	p.R132H	--	--	--	--
O2-23	co-del	p.R132H	--	--	--	--
O2-24	co-del	p.R132H	--	--	--	--
O2-25	co-del	p.R132H	--	--	--	--
O2-26	co-del	p.R132H	--	--	--	--
O2-27	co-del	--	p.R172K	--	--	--
O2-28	co-del	p.R132H	--	--	--	--
O2-29	co-del	p.R132H	--	--	--	--
O2-30	co-del	p.R132H	--	--	--	--
O2-31	co-del	p.R132H	--	--	--	--
O2-32	co-del	p.R132H	--	--	--	--
O2-33	co-del	p.R132H	--	--	--	--
O2-34	co-del	p.R132H	--	--	--	--
O2-35	co-del	p.R132G	--	--	--	--
O2-36	co-del	p.R132H	--	--	--	--
O2-37	co-del	p.R132H	--	--	--	--
O2-38	co-del	p.R132H	--	--	--	--
O2-39	co-del	--	p.R172K	--	--	--
O2-40	co-del	p.R132H	--	--	--	--
O2-41	co-del	p.R132H	--	--	--	--
O2-42	co-del	--	p.R172K	--	--	--
O2-43	co-del	p.R132H	--	--	--	--
O2-44	co-del	p.R132H	--	--	--	--

Supplemental Table 6: 1p/19q status, *IDH1*, *IDH2*, *TP53*, *ATRX* and *PTEN* mutations in individual O3 samples.

Sample ID	1p/19q status	*IDH1*	*IDH2*	*TP53*	*ATRX*	*PTEN*
O3-1	intact	p.R132H	--	p.R273C	--	--
O3-2	co-del	p.R132H	--	--	--	--
O3-3	co-del	p.R132H	--	p.T329I	--	p.P204S
O3-4	co-del	p.R132H	--	--	--	--
O3-5	co-del	p.R132H	--	--	--	--
O3-6	co-del	p.R132H	--	--	--	--
O3-7	co-del	p.R132H	--	--	--	--
O3-8	co-del	p.R132H	--	--	--	p.D52del
O3-9	co-del	p.R132H	--	--	--	--
O3-10	co-del	p.R132H	-	--	--	--
O3-11	co-del	p.R132H	--	--	--	--
O3-12	co-del	p.R132H	--	--	--	--
O3-13	co-del	--	p.R172W	--	p.G2075R	--
O3-14	co-del	p.R132H	--	--	--	--
O3-15	co-del	--	p.R172K	--	--	--
O3-16	co-del	p.R132H	--	p.T284fs	--	--
O3-17	co-del	p.R132H	--	--	--	--
O3-18	co-del	--	p.R172K	--	--	--
O3-19	co-del	p.R132H	--	--	--	--
O3-20	co-del	p.R132H	--	--	--	--
O3-21	co-del	p.R132H	--	p.R335C	--	--
